# Selective Pressures Explain Differences in Flower Color among *Gentiana lutea* Populations

**DOI:** 10.1371/journal.pone.0132522

**Published:** 2015-07-14

**Authors:** Mar Sobral, Tania Veiga, Paula Domínguez, Javier A. Guitián, Pablo Guitián, José M. Guitián

**Affiliations:** 1 Area of Ecology, University of Santiago de Compostela, 15782, Santiago de Compostela, Galicia, Spain; 2 Department of Biology, Stanford University, 385 Serra Mall, Stanford, CA, 94305, United States of America; 3 Department of Botany, University of Santiago de Compostela, 15782, Santiago de Compostela, Galicia, Spain; Kunming Institute of Botany, CHINA

## Abstract

Flower color variation among plant populations might reflect adaptation to local conditions such as the interacting animal community. In the northwest Iberian Peninsula, flower color of *Gentiana lutea* varies longitudinally among populations, ranging from orange to yellow. We explored whether flower color is locally adapted and the role of pollinators and seed predators as agents of selection by analyzing the influence of flower color on (i) pollinator visitation rate and (ii) escape from seed predation and (iii) by testing whether differences in pollinator communities correlate with flower color variation across populations. Finally, (iv) we investigated whether variation in selective pressures explains flower color variation among 12 *G*. *lutea* populations. Flower color influenced pollinator visits and differences in flower color among populations were related to variation in pollinator communities. Selective pressures on flower color vary among populations and explain part of flower color differences among populations of *G*. *lutea*. We conclude that flower color in *G*. *lutea* is locally adapted and that pollinators play a role in this adaptation.

## Introduction

Variation in flower color among plant populations may reflect adaptation to local biotic or abiotic selective pressures [1, 2, 3]. Differences in natural selection among populations may occur, for example, due to spatial variation in the interacting animal community [4]. To demonstrate local flower color adaptation it is necessary to show a relationship between color differences among populations and differences in local selective pressures. Since Darwin’s seminal studies, evolutionary biologists have studied floral variation because of the flower’s role in reproductive fitness and its potential to be selected by pollinators. Among floral traits, flower color is one of the most recognized attributes that influence pollinators [2]—flower color defines pollination syndromes associated with pollinator groups such as hummingbirds, bees or bats [5]. However, non-pollinating agents may also influence flower color diversification [2, 6, 7, 8, 9, 10, 11]. For example, pre-dispersal seed predators may be agents of selection on flower color because the adult predators oviposit during blooming, and flower color may affect their choices [12].

*Gentiana lutea* is a montane species with yellow corollas common throughout its distribution; however, at the southwestern end of its range (Iberian Peninsula, from approximately 5°30’ W, to the west) the species bears orange flowers. Corolla color in *G*. *lutea* depends upon the amount and type of carotenoids [13, 14], which is regulated by genes that control their synthesis and storage [15, 16]. Yellow/orange variation may also be due to variation in the genetically based inability to accumulate pelargonidin, (an anthocyanin pigment) which impedes the development of orange coloration [15]. However, despite flower pigments in *G*. *lutea* being genetically based, the variation in color between individuals could also be environmentally induced. But, we have analyzed whether floral color and other phenotypic traits in *G*. *lutea* are related to abiotic environmental characteristics such are soil pH, temperature, precipitation and radiation, and have found no relationships between these factors and flower color in this species (unpublished data). Thus, floral color variation does not appear to be the result of environment-dependent phenotypic plasticity or adaptation to local abiotic conditions. However, to confirm that color variation among individuals is determined by genetic differences among them, it would be necessary to demonstrate either heritability of color within populations or genetic differences among differently colored populations. Genetic differences among populations (using molecular markers) have been found [17], but it is not known if those genetic differences correlate with differences in flower color.

Flower color in *G*. *lutea* affects plant reproductive success through the action of pollinators and seed predators in a population [18]. Pollinators make more visits to yellow flowers, while seed predators oviposit more often in individuals with orange flowers [18]. Since pollinators are mutualists and seed predators are antagonists, they both increase the reproductive output of yellow flowering individuals in that particular population [18]. Thus, pollinators and seed predators show flower color preferences which affect reproductive success of *G*. *lutea*, therefore they might influence color variation among populations.

The most abundant *G*. *lutea* pollinators, *Bombus terrestris* and *B*. *pratorum* [18], exhibit low sensitivity to the color red [19, 20], although they potentially can still distinguish between orange and yellow by judging the gray-scale contrast. However, there are other pollinator species, such as *Bombus lapidarius* with sensitivity to the color red [19, 21] which also interact with *G*. *lutea* plants. Therefore, the composition of pollinator communities might affect the selective pressures exerted on *G*. *lutea* flower color and ultimately affect flower color variation among populations via local adaptation.

Demonstrating variation in selective pressures among populations and analyzing its relationship with the spatial variation of plant traits is a powerful approach for measuring the effect of natural selection in the wild [22, 23]. We first studied the possible role of pollinators and seed predators as selective agents driving color variation among populations by (1) analyzing if flower color influenced the interaction of plants with pollinators and seed predators, and (2) investigating whether flower color variation among populations is related to variation in pollinator communities. Second, we explored whether local selective pressures vary among populations and explain geographic variation in flower color by (3) examining how the effect of flower color on plant fitness varies among populations, and (4) testing whether local selective pressures explain the flower color variation among populations. We show that natural selection is responsible, at least in part, for the color differences among populations of *Gentiana lutea*.

## Materials and Methods

*Gentiana lutea* is an endangered plant species listed in Annex V of the EU Habitats Directive; collection of the rhizome is regulated. Since we did not collect any rhizomes, no permits were required. For access to public land, the Territorial Service of Environment of León issued permission following the relevant laws (Art. 36 and 37.1, Law 8/1991, 10 of May; Art 72.1 of the P.O.R.N. of Parque Regional de Picos de Europa en Castilla y León; Art 45.4, Law 42/2007, 13 of December). For access to private land, we obtained permission from the land owners.

### Species

The Yellow Gentian (*Gentiana lutea* L. Gentianaceae) is a long-lived species distributed across montane and subalpine habitats of central and southern Europe, from Anatolia to the Iberian Peninsula [24]. Basal leaves are lanceolate-elliptic to broadly ovate. During early summer (June), the rhizome develops one to three flowering unbranched stout stems growing up to 1.40 m tall. The stem develops about 80 pedicellate flowers on 4 to 8 nodes, each node composed by two pseudo-whorls. Flowers are actinomorphic; corollas are deeply divided with (4–7) nearly free petals and (4–7) free stamens. Flower color varies among individuals as a continuum from brick-orange to pale-yellow. The fruit is an ovoid capsule that contains around 75 winged seeds of 4 mm diameter that are dispersed by wind [25]. It attracts a wide range of pollinators, mostly bumblebee species [26]. Lepidoptera larvae prey upon *G*. *lutea* seeds prior to dispersal [18].

### Field procedures

**Fig 1:**
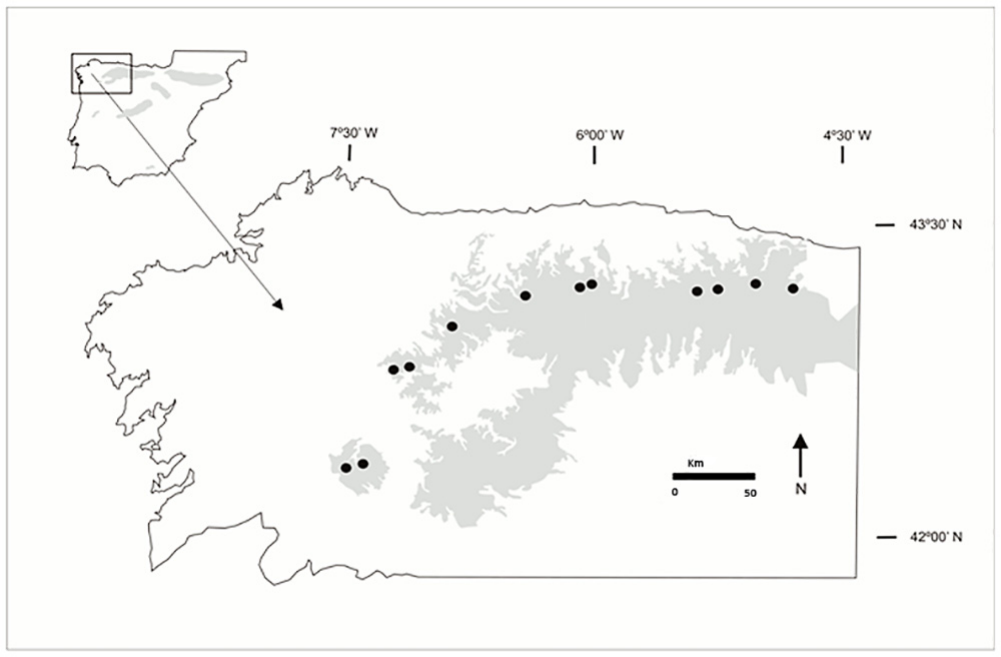
Location of the *Gentiana lutea* populations sampled. The shaded region indicates the distribution of *G*. *lutea* in the Iberian Peninsula.

We studied 12 *G*. *lutea* populations in two consecutive years, 2010 and 2011 (three populations, San Mamede, Loureses and Ponton, were studied only in 2011). In order to properly test the spatial variation of selection, we made an effort to sample a large number of populations and cover a wide geographical range. The study area covered the western end of the species distribution in the Cantabrian Mountains. Populations were chosen haphazardly along a longitudinal transect from San Mamede (7°30’ W, west) to San Glorio (4°45’ W, east), 230 km apart (Fig 1). Longitudinal coordinates were obtained for each population with a GPS (Garmin eTrex Vista). In June, when the stem was developed but before blooming started, we haphazardly chose in each population between 20 to 45 plants per year. The relatively small number of plants sampled per population was due to the very short flowering time in *G*. *lutea* (only several days), the trade-off between number of plants sampled per population and the number of populations studied, and the small number of flowering individuals in some populations.

#### Flower color and other plant traits

In July 2010, during blooming, we measured flower color on ten randomly chosen flowers per plant. In 2011, we measured color on only three flowers per plant because we found that the coefficient of variation for flower color within plants was asymptotic, reaching a plateau after 3 flowers. On each flower, immediately after collection, we measured one fresh petal on its adaxial surface at the midpoint of the petal. Each petal was measured three times. Final color spectrum data for individual plants came from the mean of these three measurements per petal for 10 petals belonging to 10 different flowers in 2010 (30 floral color data per plant in 2010) and three measurements for each of three petals belonging to 3 different flower is 2011 (nine color data per plant in 2011). We measured floral color in a total of 2,711 flowers belonging to 504 plants across 12 *G*. *lutea* populations. Color spectra were obtained with a USB2000+ fiber optic spectrometer (Ocean Optics, Inc., Dunedin, FL) under the following conditions: each sampling session, calibration was done with a white standard; the angle of measurements was fixed at 45° relative to petal surface; the probe was shielded to all ambient light, with the only light provided by a deuterium tungsten halogen source (DT-MINI-2-GS). SpectraSuite software was used to process spectra measurements (Ocean Optics, Inc., Dunedin, FL, USA).

We focused our study of flower color to the visual range of the spectrum. Although UV light can be important to pollinator attraction, we found in a pilot study that there were no differences in UV light reflectance between plants with different flower color (see S1 Text for the methodology employed). Spectra from 380 to 780 nm at 5 nm intervals were transformed to obtain three color variables following the CIELab colorimetric system [27]. This system defines color by the following factors: *L*, which measures lightness and varies from black (0) to white (100); *a*, which ranges from green (-110) to red (110); and *b*, which ranges from blue (-110) to yellow (110). The three variables taken together represent a three dimensional sphere-shaped space where color changes as a continuous variable; it is known as the chromaticity CIELab space. The parameters employed were the CIE 1964 standard colorimetric observer (10° visual field) and the CIE standard illuminant A.

Beside floral color, on each plant we measured petal length, petal width, length of the longest basal leaf, and counted the number of flowers. Three petals per flower were measured on three randomly chosen flowers per plant to obtain the average value for plant petal length and width. We measured petal length from the insertion until the tip and petal width on the mid part; we used a caliper to the nearest 0.1 mm. We measured the length of the longest basal leaf from the insertion to the tip with a measuring tape to the nearest 1 mm.

#### Plant pollinator visitation rate and escape from seed predation

We determined the identity and abundance of pollinators for each plant by counting all insects known to be *G*. *lutea* pollinators [26] visiting flowers and making contact with the anthers and stigmas. We made 10 censuses of 1 minute per plant during 2010 and 10 censuses of 2 minutes per plant in 2011; a total of 130 h of censuses for 466 plants. Censuses were done between 10h and 19h (Greenwich Mean Time) with temperatures ranging between 12°C and 26°C, and no windy conditions or rain. We considered pollinator visitation rate as the number of total visits per minute for each plant. We captured some specimens for further identification; we assessed the pollinator community using distinguishable pollinator groups which were: *Bombus terrestris* and *B*. *lucorum*; *Bombus hortorum* and *B jonellus*; *Bombus pratorum* and *B*. *soroeensis*; *Bombus wurflenii* and *B*. *lapidarius*; *Bombus mesomelas*; *B*. *pascuorum*; subgen. *Psithyrus*; and *Apis* spp.

In both years, in August, when fruits were ripe but not opened yet, we counted the total number of fruits and examined seed predation in each of 431 plants. From ten randomly selected fruits on each plant, we counted the number of fruits affected by seed predators (showing signals such as holes or rotting). We obtained the rate of the escape from seed predation as the percentage of fruits that were not affected by predators.

#### Plant seed production

We collected between seven to ten fruits per plant and counted the number of seeds within them. We estimated the total seed production of each plant as the average seed number per fruit, multiplied by the total number of fruits on each plant. We examined seed production for 3,644 fruits belonging to 383 plants. We used seed production per plant as a proxy for fitness; in long-lived species (such as *G*. *lutea*) seed production, and other measures of reproductive success, are conventional surrogates for fitness as measuring total fitness is often not possible (see [28]).

### Statistical analyses

We reduced the three color variables (*L*, *a*, and *b*) using principal component analysis. The first principal component (PC1) represented 63% of variance in *a*, *b* and *L* (eigenvalue = 1.89; 2,711 flowers); thus, PC1 is considered the flower color variable in further analysis. Note that we have additionally performed the analyses using both PC1 and PC2 (jointly explaining 95% of variation in flower color) and results remained the same (results not shown). Thus, we present results using only PC1 as a color descriptor for the sake of simplicity. Low scores of the PC1 indicate dark-orange color and high scores indicate bright-yellow color since factor coordinates of the original variables on PC1 were L (brightness) = 0.686, *a* (red related component) = -0.360, and *b* (yellow related component) = 0.631.

Although we focused on flower color, we included other phenotypic traits in the following analyses to control for their possible effects on pollination, seed predation and seed production, or their correlation with flower color. We performed Pearson correlations for the traits under study.

We followed the approach proposed by Herrera et al. [22] to test for natural selection driving local adaptation and the role played by the animal community. To this end, we performed the following analyses (using the SPSS for windows version 20, IBM SPSS Statistics 20).

#### Flower color effect on pollination and seed predation; among population variation

Pollinator visitation rate and escape from seed predation were dependent variables in two independent generalized linear models in which the explanatory variables were plant traits (flower color, petal width, petal length, leaf length and number of flowers), population, year, and interactions between population and plant traits. We considered population as a fixed factor because we focus on differences among populations, which follow a longitudinal gradient. Error distributions and link functions were chosen following the AICc criterion [29]. Pollinator visitation rate was fitted to a gamma distribution with a logarithmic link function; escape from seed predation was fitted to a Poisson distribution with a logarithmic link function. Plant traits were centered (we subtracted the average) to avoid colinearity.

#### Flower color variation and differences in pollinator communities

In order to analyze the relationship between pollinator communities and the flower color at the population level we built a dissimilarity matrix on the pollinator assemblage for each population and year following the procedures described in Manzaneda and Rey [30]. A dissimilarity matrix of the assemblage of pollinator groups was built by pairwise dissimilarity coefficients (1-PS) in the relative abundance of the groups. PS coefficients (proportional similarity Renkonen index) were assessed from the relative abundance (estimated from frequency of interaction rather than from absolute or ambient abundance, in each population). PS for a pair of populations a and b is:

$$\sum_{i=n}^{n}\text{min }(p_{ai},\;p_{bi})\;$$

where n is the number of species, p_*ai*_ is the relative abundance of species i in population a, and p_*bi*_ is the relative abundance of species i in population b.

The PS index ranges from 0 (standing for maximum dissimilarity, i.e. no common groups between a and b) to 1 (standing for maximum resemblance, i.e. identical species composition between a and b). We also calculated one matrix for flower color differences between populations in which the entries were the absolute differences in color means between populations. We tested the correlation between both matrices (we lacked pollinator community data for the population of Queixa in 2010, and thus we analyzed eight populations in 2010 and 12 populations in 2011, resulting in 190 comparisons) by the Mantel test (Manteltester 1.0; [30]).

#### Variation of selective pressures among populations

We analyzed the variation among populations in the relationship between plant traits and fitness—following the procedure described in Herrera et al. [22]. We built a generalized linear model in which seed production was the dependent variable. We considered the number of seeds produced by each plant as a proxy for plant fitness, which is a conventional surrogate in long-lived species [28]. Independent variables included population, year, plant traits (flower color, petal width, petal length, leaf length and number of flowers), and two-way interactions between population and each plant trait. We included population as a fixed factor because populations follow a longitudinal gradient. Error distribution and link function were chosen following the AICc criterion [29]. Seed production was fitted to a gamma distribution with a logarithmic link function.

#### Effect of selective pressures on the spatial variation of flower color

If divergence among populations in flower color is currently being caused by divergent selection, then we would expect the selection differential on flower color to be correlated with flower color among populations. Specifically, populations with mostly yellow flowering individuals should exhibit selection favoring yellow flowering plants, while populations with primarily orange flowering individuals should exhibit selection favoring orange flowering individuals.

To test this, we first calculated the selection differentials (S) on flower color (the standardized coefficient of a simple regression of flower color on relative fitness [31, 32] for each population and year, in order to use them as predictors of flower color variation among populations. Selection differentials estimate the strength and direction of selection due to the combined effects of all selective forces. They account for both the positive effect of mutualistic pollinators and the negative effect of antagonistic seed predators. Additionally, selection differentials include the effect of indirect selection due to selection on correlated traits.

We designed a generalized linear model in which the population mean flower color each year was the response variable explained by the following independent variables: the year (as a fixed factor) with the selection differential on flower color (S), geographical longitude and averages of population’s plant traits (petal width, petal length, leaf length and number of flowers) as covariates. No interaction terms were included. Error distribution and link function were chosen following the AICc criterion [29]. Flower color fitted a normal distribution with a logarithmic link function.

We additionally calculated the proportion of variance explained by selection differentials by running an univariate analysis of variance with flower color as the response variable (log-transformed) and the geographical longitude, year, and selection differentials as the explanatory factors. The proportion of variance explained was calculated from the type III sum of squares.

## Results

Flower color is correlated with petal width and with the number of flowers: orange plants have fewer flowers with wider petals while yellow plants have more flowers with narrower petals. In general, plants with longer leaves had more and bigger flowers. These correlations show the necessity of including as many plant traits as possible in the analyses of selection on flower color so that we can discard spurious effects due to phenotypic correlations (S1 Table).

### Flower color effect on pollination and seed predation: among population variation

The mean number of pollinator visits per minute per plant was 1.24 (± 1.54 S.D.); we recorded a total of 7,016 pollinator visits to 466 plants during 130 h of censuses. Mean escape from seed predation rates per plant averaged 65.2% ± 31.7% S.D. Flower color affected pollinator visits and this effect varied among populations (as indicated by the population * color interaction in Table 1). There were populations in which yellow flowering individuals received more visits, whereas in other populations orange flowering plants received more visits or pollinators did not show any preference (not shown). We did not detect an effect of flower color on seed predation. Petal length and number of flowers also affected pollinator visitation rate and escape from seed predation, but we did not find any spatial variation on these effects (Table 2). Thus, phenotypic traits affected both the chance of plants being visited by pollinators and their chance of being visited by seed predators. Interestingly, only the effect of flower color on pollinator visits varied among populations.

**Table 1:** Results of the GzLMs analyzing the variation among populations in the influence of flower color on pollinator visitation rate in G. lutea. N = 443. In bold are effects with p values < 0.05. Factor codes: PL, petal length; PW, petal width; LL, leaf length; FN, number of flowers; Pop, population.

Dependent	Factor	s.e.	Chi-square	d.f.	*p*
Pollinator visitation rate	Flower color	0.187	0.0	1	0.925
	**PL**	**0.084**	**7.9**	**1**	**0.005**
	PW	0.305	1.2	1	0.267
	**FN**	**0.006**	**8.2**	**1**	**0.004**
	LL	0.004	2.2	1	0.136
	**Pop**		**123.7**	**11**	**0.000**
	**Year**		**16.5**	**1**	**0.000**
	Pop * year		**43.6**	**8**	**0.000**
	Pop * flower color		**25.0**	**11**	**0.009**
	Pop * PL		9.0	11	0.617
	Pop * PW		11.3	11	0.414
	Pop * FN		11.5	11	0.403
	Pop * LL		11.3	11	0.421

**Table 2:** Results of the GzLMs analyzing the variation among populations in the influence of flower color on escape from seed predation in G. lutea. N = 406. In bold are effects with p values < 0.05. Factor codes: PL, petal length; PW, petal width; LL, leaf length; FN, number of flowers; Pop, population.

Dependent	Factor	Coefficient	s.e.	Chi-square	d.f.	*p*
Escape from seed predation	Flower color	0.016	0.105	0.0	1	0.950
	**PL**	**0.051**	**0.048**	**7.4**	**1**	**0.007**
	PW	-0.176	0.196	2.5	1	0.114
	**FN**	**-0.003**	**0.003**	**5.3**	**1**	**0.021**
	LL	0.000	0.002	1.2	1	0.278
	**Pop**			**65.4**	**11**	**0.000**
	Year			2.8	1	0.095
	Pop * year			**77.4**	**8**	**0.000**
	Pop * flower color			8.1	11	0.706
	Pop * PL			13.0	11	0.293
	Pop * PW			11.3	11	0.417
	Pop * FN			15.1	11	0.179
	Pop * LL			7.3	11	0.771

### Flower color and pollinator community

**Fig 2:**
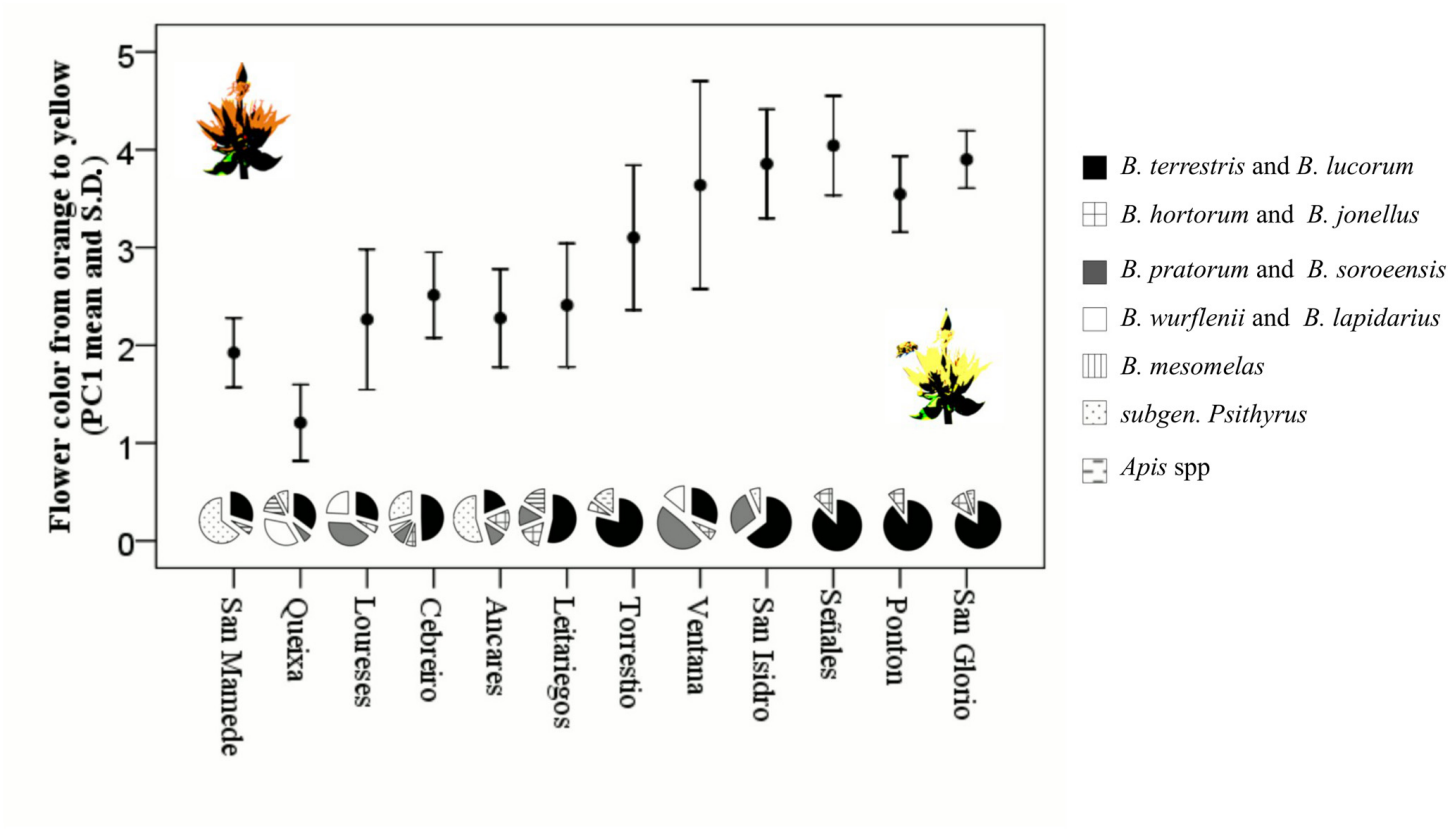
Flower color and pollinator community composition for the 12 *Gentiana lutea* populations studied. Average flower color and standard deviation are shown for each population. Pie charts represent the composition of pollinator community within each population. For the sake of simplicity only pollinator guilds representing at least 5% of the visitation in a given population were illustrated. See S2 Table for exact number of visits per group.

**Fig 3:**
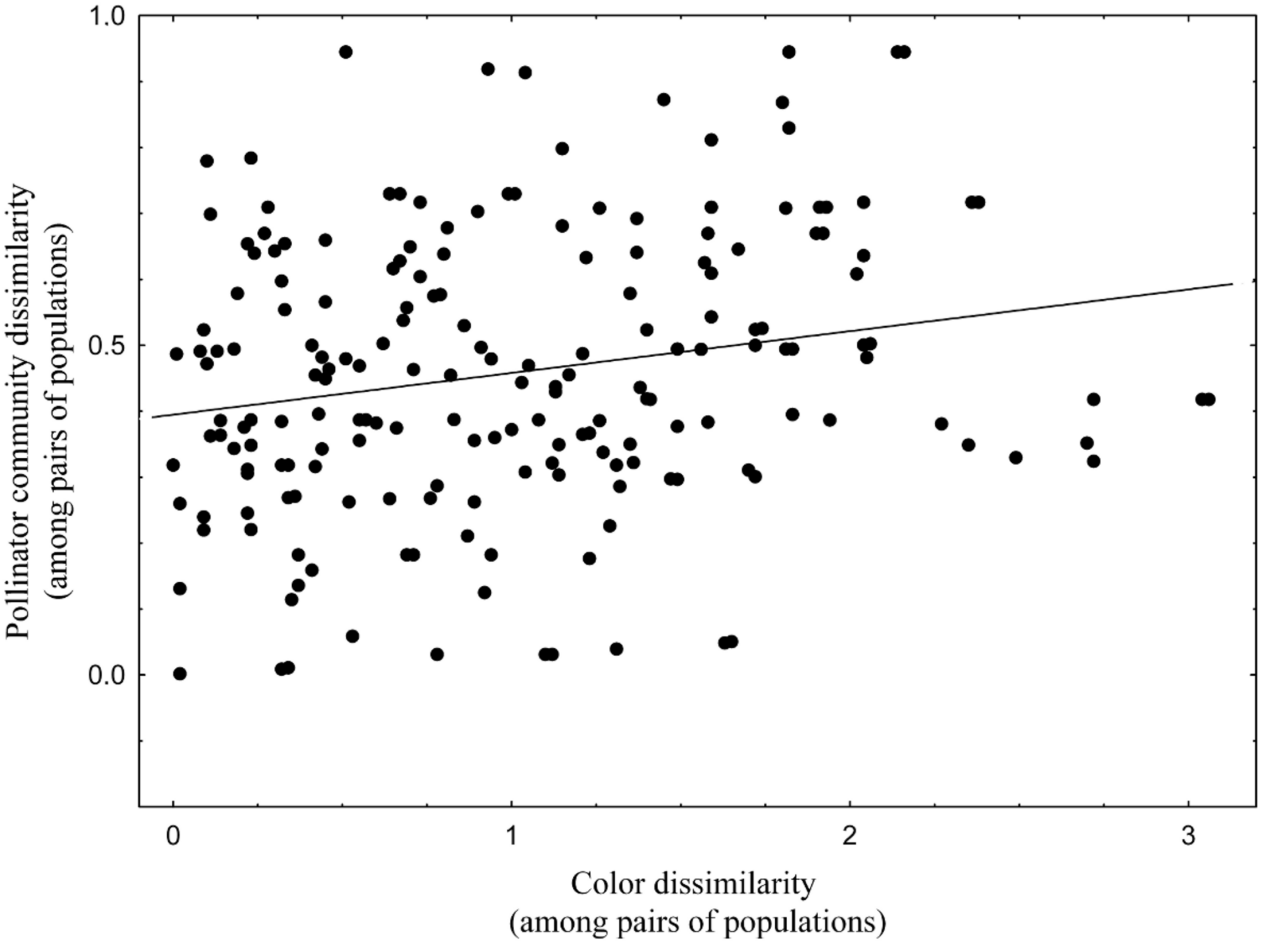
Pollinator community and color variation. Relationship between flower color dissimilarity among populations and pollinator community dissimilarity among populations (8 populations in 2010, 12 populations in 2011; N = 190, r = 0.208, *p* < 0.05).

*Gentiana lutea* plants received visits from bumblebees (79%), cuckoo bumblebees (20%), and honeybees (1%). Within bumblebees we found ten species: *B*. *terrestris*, *B*. *lucorum*, *B*. *pratorum*, *B*. *soroeensis*, *B*. *lapidarius*, *B*. *wurflenii*, *B*. *hortorum*, *B*. *jonellus*, *B*. *mesomelas*, and *B*. *pascuorum* (S2 Table and S1 Dataset). As shown by the Mantel test, differences in pollinator communities correlate to differences in flower color among populations; when compared pairwise, the dissimilarity between populations in the pollinator community is positively correlated with dissimilarity in flower color (r = 0.208, *p* < 0.05; Figs 2 and 3).

### Variation of selective pressures among populations

The number of flowers affected seed production, but its effect did not vary among populations (as shown by the non-significant flower number * population interaction, Table 3).

**Table 3:** Results of the GzLM analyzing the variation among populations in the selective pressures on flower color. N = 350. In bold are effects with *p* values < 0.05. Factor codes: PL, petal length; PW, petal width; LL, leaf length; FN, number of flowers; Pop, population.

Dependent	Factor	Coefficient	s.e.	Chi-square	d.f.	*p*
Seed production	**Flower color**	**0.100**	**0.227**	**4.2**	**1**	**0.041**
	PL	0.064	0.098	2.5	1	0.111
	PW	0.065	0.461	0.4	1	0.532
	**FN**	**0.008**	**0.007**	**23.9**	**1**	**0.000**
	LL	0.001	0.004	2.3	1	0.127
	**Pop**			**117.0**	**11**	**0.000**
	Year			2.0	1	0.156
	Pop * year			4.5	7	0.724
	Pop * flower color			**21.1**	**11**	**0.032**
	Pop * PL			8.4	11	0.673
	Pop * PW			7.5	11	0.759
	Pop * FN			7.9	11	0.725
	Pop * LL			15.8	11	0.148

Flower color influenced seed production and selective pressures on flower color varied among populations. The magnitude and direction of selection on flower color within populations is indicated by the slope of the relationship between seed production and flower color. We detected spatial variation in the relationship between flower color and seed production as indicated by the significant color * population interaction. Populations differed in magnitude, direction and type of selection on color (see in S3 Table significant selection coefficients for each of the studied populations).

### Effect of selective pressures on the spatial variation of flower color

Selection acts within populations; if selective pressures differ among populations, we might expect phenotypic differences among them. Thus, a correlation between selection differentials and flower color would imply ongoing divergent selection in flower color among populations.

**Fig 4:**
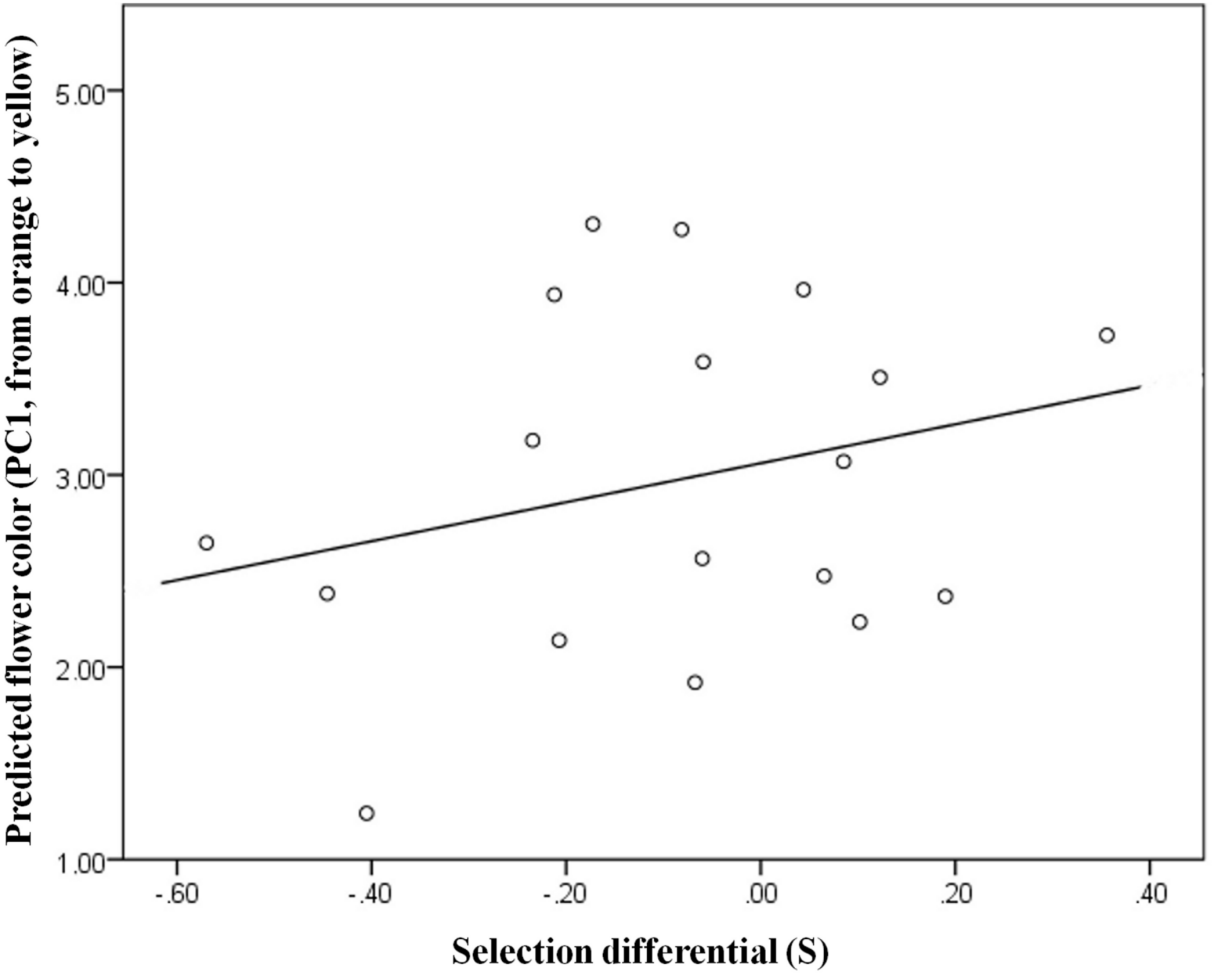
Relationship between selection differentials on flower color and flower color variation among populations. N = 18 (12 populations, some of which were studied across two years).

Selection differentials explained part of the flower color variation among populations: plants growing in populations where there is selection in favor of yellowness bear yellow flowers; plants growing in populations where there is selection in favor of orange coloration tended to bear orange flowers (Table 4; Fig 4). Note that in this study negative selection differentials indicate selection favoring orange flowering individuals while positive differentials indicate selection favoring yellow flowering individuals. The longitudinal coordinate explained part of the flower color variation among populations: orangeness increases westward (Table 4; Fig 4). Flower color differences among populations are explained mostly by the geographical longitude (45%), followed by the selection differential, which explains 6% of the variation in flower color among populations.

**Table 4:** Results of the GzLM model analyzing the effect of variation in selection differentials on flower color variation among populations of *G*. *lutea*. N = 18. In bold are effects with *p* values < 0.05.

Dependent	Factor	Coefficient	s.e.	df	*p*
Flower color	**Selection differential (S)**	**0.302**	**0.115**	**1**	**0.007**
	**Longitudinal coordinate**	**-0.217**	**0.007**	**1**	**0.005**
	Flower number	0.001	0.002	1	0.513
	Petal width	-0.094	0.082	1	0.252
	Petal length	0.016	0.003	1	0.545
	Leaf length	0.000	0.026	1	0.866
	Year	0.090	0.106	1	0.398

## Discussion

Our results support the hypothesis that natural selection drives, to some extent, the geographic structure of flower color variation among populations in this species, most likely through the action of pollinators. We base our conclusion on four findings: (*i*) flower color affects pollinator visitation rates differently for different populations (Table 1), (*ii*) differences in pollinator assemblages correlate with flower color differences in populations (Figs 2 and 3), (*iii*) selection pressures on color vary among populations (Table 3), (*iv*) variation in selection explains differences in population flower color (Table 4; Fig 4). Showing that flower color varies among populations of *G*. *lutea*, and that this variation is explained by local selective pressures (while also identifying the agents of selection) increases our understanding of the adaptive nature of flower color. Despite the well-studied relationship between pollinators and floral variation, only a few studies have provided evidence that pollinators have a role in shaping the geographical structure of flower color [33, 34].

Our data indicate that selection shapes at least part (6%) of the variation in flower color. Phenotypic selection analyses have certain limitations. On the one hand, the evolutionary effects of the action of selection may be influenced by other selective pressures, such as those exerted by the seed environment during germination or by conditions during growth and survival. On the other hand, if phenotypes and fitness covary with environment, measurements of selection might be biased [35, 36]. However, our research not only investigates the existence of selection, but tests its effects across spatial phenotypic variation. Despite the above-mentioned limitations we found a relationship between local selection and local flower color.

Our results support the hypothesis that flower color variation may arise not only from selection on qualitative variation but from selection on quantitative variation as well. Selection on flower color may occur on genes that produce qualitative changes [2]. Nevertheless, the evolution of floral coloration might additionally involve more subtle, quantitative color adaptation [37]. Frey [9] and Caruso [7] found selection on quantitative variation in flower color with floral redness favored in *Claytonia virginica*, and flower brightness favored in *Lobelia siphilitica*, respectively. More recently, Renoult [37] found selection on quantitative flower color variation in *Centaurea cyanus* and, more importantly, they demonstrated that the visual system of bumblebees allowed for preferences based on quantitative variation in flower color. Here, we found that pollinators perceive and react to quantitative variation in flower color of *G*. *lutea* causing different selective pressures across sites.

We found different sets of insect species pollinating *G*. *lutea* at different sites. Insect communities might be determined by many factors such as latitude, altitude, or plant communities present nearby. Our results support the hypothesis that differences among populations in selective pressures on flower color are related to differences in the pollinator communities. This is likely because pollinator species have dissimilar preferences towards floral traits such as color due to differences in their visual systems [38], which cause differences in their abilities to detect particular colors [39]. The species *Bombus terrestris* manifests low sensitivity for detecting red colors [19, 20] and is more abundant in yellow-flowered populations than in orange-flowered populations (Fig 2, S2 Table). Additionally, *Bombus lapidarius* shows sensitivity for red signals [19, 21], and is abundant in some orange-flowered populations and scarce in yellow-flowered populations (Fig 2; S2 Table). Thus, variation in the pollinator assemblage seems to cause different selective pressures among populations and affect flower color variation among *G*. *lutea* populations.

Seed predators were not found to be selective agents driving color variation among populations in *G*. *lutea*, despite exerting selection on flower color within a population [18]. Seed predators are, however, selective agents on other traits such as petal length and number of flowers. Since the number of flowers correlate to flower color (S1 Table), flower color may be subject to indirect selection by seed predators. Apart from the spatial variation of selection driven by the animal community, other non-mutually exclusive hypotheses might also explain the geographic structure of flower color in this species. These hypotheses include selective pressures [6, 40] or plasticity in flower color related to abiotic conditions [41, 42, 43]—although soil pH, radiation, temperature and precipitation do not affect flower color variation in *G*. *lutea* (unpublished data), historical factors such as genetic isolation and diversification due to the cyclical climatic changes in the Quaternary, [44, 45, 46] and genetic drift.

Our study relies on observational data; to test the causal relationship between selective pressures and phenotypes we would need to develop experiments such as reciprocal transplants [47]. Artificial conditions created during experiments, however, may influence plant and animal responses, and cause them to differ from those in natural conditions. We advocate for the importance of observational studies developed in the wild as an essential contribution to understanding nature. Assessing the impact of selection on phenotypic variation among populations is a powerful approach to identify local adaptation in natural conditions, especially when studying long-lived organisms at broad geographical scales [22, 23]. We show that natural selection—most likely exerted by pollinators—explains flower color variation among populations of *Gentiana lutea*.

## Supporting Information

S1 Dataset Plant data and pollinators visits per population. (XLS)

S1 Table Phenotypic plant trait correlations. Correlation coefficients (r) are indicated above the diagonal, and the *p* values are indicated below the diagonal. In bold are correlations with *p* < 0.05, N = 429.
(DOC)

S2 Table Pollinator assemblage across *Gentiana lutea* populations. We provide the number and percentage (in parentheses) of visits to *G*. *lutea* flowers of different morphological groups; some species were grouped because differentiation was difficult during census. We pooled data from 2010 and 2011; we recorded a total of 7,016 visits on 466 plants, during 130 hours of census. We included two diversity indices: S, the species richness, obtained from the number of different groups present in each population; H’, the Shannon-Weaver index which considers both species richness and abundance of species; the higher the values, the higher the diversity.
(DOC)

S3 Table Population traits and coefficients of selection on flower color. We calculated the mean and standard deviation (in parentheses) of the phenotypic traits, and selection coefficients on flower color, in each population. We show the standardized significant (*p* < 0.05) coefficients of selection on flower color [30]. We used as covariates: petal length, petal width, leaf length, the number of flowers and the height of the stalk. We obtained the total selection differential (S), direct selection gradients (β), and quadratic (γ_ii_) and correlational selection gradients (γ_ij,_ γ_ih_; i = flower color, j = petal width, h = number of flowers). Note that the quadratic selection coefficients are correctly assessed by doubling the standardized coefficient obtained by the regression.
(DOC)

S1 Text UV light reflectance. (DOC)
