# Supplementary material for: Selective Pressures Explain Differences in Flower Color among Gentiana lutea Populations
Source: PLoS One. 2015 Jul 14;10(7):e0132522. doi: 10.1371/journal.pone.0132522 (PMC4501686; doi:10.1371/journal.pone.0132522)
Supplement: S1 Table — Correlation coefficients (r) are indicated above the diagonal, and the p values are indicated below the diagonal. In bold are correlations with p < 0.05, N = 429. (DOC) [file pone.0132522.s002.doc]

**S1. Table.** Phenotypic plant trait correlations. Above diagonal the correlation coefficient (r), bellow diagonal the P values. In bold are correlations with *p* < 0.05. N = 429.

|  | Flower color | Petal length | Petal width | Nº of flowers | Leaf length |
| --- | --- | --- | --- | --- | --- |
| Flower color | — | **-0.118** | **-0.493** | **0.166** | 0.005 |
| Petal length | 0.014 | — | **0.524** | -0.007 | **0.270** |
| Petal width | 0.000 | 0.000 | — | -**0.140** | **0.115** |
| Nº of flowers | 0.001 | 0.882 | 0.004 | — | **0.230** |
| Leaf length | 0.913 | 0.000 | 0.017 | 0.000 | — |
