# Supplementary material for: Selective Pressures Explain Differences in Flower Color among Gentiana lutea Populations
Source: PLoS One. 2015 Jul 14;10(7):e0132522. doi: 10.1371/journal.pone.0132522 (PMC4501686; doi:10.1371/journal.pone.0132522)
Supplement: S2 Table — We provide the number and percentage (in parentheses) of visits to G. lutea flowers of different morphological groups; some species were grouped because differentiation was difficult during census. We pooled data from 2010 and 2011; we recorded a total of 7,016 visits on 466 plants, during 130 hours of census. We included two diversity indices: S, the species richness, obtained from the number of different groups present in each population; H’, the Shannon-Weaver index which considers both species richness and abundance of species; the higher the values, the higher the diversity. (DOC) [file pone.0132522.s003.doc]

**S2. Table. Pollinator assemblage across *Gentiana lutea* populations.** We provide the number and percentage (among parenthesis) of visits to *G. lutea* flowers of different morphological groups; some species were joined because differentiation was difficult during census. We joint data of 2010 and 2011; we recorded a total of 7016 visits on 466 plants, during 130 hours of censuses. We included two diversity indexes: S, the species richness, obtained from the number of different groups present in each population; H’ is the Shannon-Weaver index which considers both, species richness and abundance of species; the higher the values, the higher the diversity.

| Population | *B. terrestris, B. lucorum* | *B. hortorum, B. jonellus* | *B. pratorum, B. soroeensis* | *B. wurflenii, B. lapidarius* | *B. mesomelas* | *B. pascuorum* | subgen. *Psithyrus* | *Apis s*pp | S | H’ |
| --- | --- | --- | --- | --- | --- | --- | --- | --- | --- | --- |
| San Mamede | 189 (28) | 18 (3) | 6 (1) | 11 (2) | 36 (5) | 0 | 408 (61) | 0 | 6 | 1.48 |
| Queixa | 304 (35) | 5 (1) | 52 (6) | 323 (37) | 123 (14) | 0 | 70 (8) | 3 (0) | 8 | 2.03 |
| Loureses | 226 (29) | 37 (5) | 317 (41) | 187 (24) | 0 | 7 (1) | 4 (1) | 0 | 7 | 1.85 |
| Cebreiro | 182 (49) | 27 (7) | 35 (9) | 17 (5) | 5 (1) | 0 | 107 (29) | 2 (1) | 8 | 1.94 |
| Ancares | 259 (18) | 205 (14) | 168 (11) | 51 (3) | 0 | 15 (1) | 768 (52) | 0 | 7 | 1.92 |
| Leitariegos | 199 (51) | 57 (15) | 50 (13) | 10 (3) | 63 (16) | 2 (1) | 5 (1) | 5 (1) | 8 | 2.04 |
| Torrestio | 112 (78) | 8 (6) | 3 (2) | 0 | 0 | 0 | 0 | 21 (15) | 4 | 1.04 |
| Ventana | 100 (30) | 19 (6) | 156 (47) | 45 (14) | 11 (3) | 0 | 0 | 0 | 5 | 1.82 |
| San Isidro | 512 (57) | 23 (3) | 221 (25) | 11 (1) | 21 (2) | 11 (1) | 58 (6) | 38 (4) | 8 | 1.83 |
| Señales | 407 (80) | 63 (12) | 12 (2) | 0 | 0 | 10 (2) | 14 (3) | 2 (0) | 4 | 1.04 |
| Ponton | 139 (82) | 21 (12) | 4 (2) | 0 | 0 | 0 | 6 (4) | 0 | 4 | 0.91 |
| San Glorio | 309 (75) | 43 (10) | 4 (1) | 4 (1) | 10 (2) | 8 (2) | 12 (3) | 20 (5) | 8 | 1.38 |
| Total | 2938 (43) | 526 (7) | 1028 (15) | 659 (9) | 269 (4) | 53 (1) | 1452 (21) | 91 (1) | 8 |  |
