# Supplementary material for: Selective Pressures Explain Differences in Flower Color among Gentiana lutea Populations
Source: PLoS One. 2015 Jul 14;10(7):e0132522. doi: 10.1371/journal.pone.0132522 (PMC4501686; doi:10.1371/journal.pone.0132522)
Supplement: S3 Table — We calculated the mean and standard deviation (in parentheses) of the phenotypic traits, and selection coefficients on flower color, in each population. We show the standardized significant (p < 0.05) coefficients of selection on flower color [30]. We used as covariates: petal length, petal width, leaf length, the number of flowers and the height of the stalk. We obtained the total selection differential (S), direct selection gradients (β), and quadratic (γii) and correlational selection gradients (γij, γih; i = flower color, j = petal width, h = number of flowers). Note that the quadratic selection coefficients are correctly assessed by doubling the standardized coefficient obtained by the regression. (DOC) [file pone.0132522.s004.doc]

**S3. Table.** Population´s traits and coefficients of selection on flower color. We calculated the mean and standard deviation (among parenthesis) for the phenotypic traits and selection coefficients on flower color, in each population. We show the standardized significant (*p* < 0.05) coefficients of selection on flower color. To calculate the standardized selection coefficients, we followed de procedures described in Lande [25]. We obtained the total selection differential (S), direct selection gradient (β; we used as covariates: petal length, petal width, leaf length, the number of flowers and the height of the stalk); and quadratic (γ_ii_) and correlational selection (γ_ij_, γ_ih_; i = flower color, j = petal width, h = number of flowers). Note that the quadratic selection coefficients are correctly assessed by doubling the standardized coefficient obtained by the regression.

| Population | Flower color (PC1) | Leaf length (mm) | Petal length (mm) | Petal width (mm) | Number of flowers | Standardized coefficients of selection on flower color |
| --- | --- | --- | --- | --- | --- | --- |
| San Mamede | 2.5 (0.4) | 238 (41.7) | 25.1 (1.5) | 6.4 (0.6) | 77.3 (21.2) |  |
| Queixa | 1.2 (0.4) | 231 (41.5) | 26.1 (2.7) | 6.8 (1.2) | 74.3 (25.1) | S = -0.75 |
| Loureses | 2.4 (0.7) | 236 (62.6) | 27.0 (2.7) | 7.4 (0.7) | 77.6 (22.3) | γ_ii_ = 1.95; γ_ij_ = -1.28 |
| Cebreiro | 2.5 (0.4) | 253 (45.6) | 24.2 (2.1) | 5.8 (1.1) | 92.6 (29.0) | γ_ij_ = -0.40 |
| Ancares | 2.3 (0.5) | 274 (40.7) | 27.2 (2.4) | 6.0 (0.8) | 89.1 (19.4) | β = -0.21 |
| Leitariegos | 2.4 (0.6) | 239 (44.0) | 22.7 (2.8) | 5.4 (0.9) | 76.1 (22.8) | γ_ii_ = -1.13; γ_ih_ = -1.44 |
| Torrestio | 3.1 (0.7) | 244 (47.8) | 26.4 (3.1) | 5.7 (0.9) | 86.6 (26.4) | γ_ih_ = 0.20 |
| Ventana | 3.5 (0.8) | 259 (43.3) | 26.5 (3.7) | 5.2 (1.2) | 85.8 (30.0) | β = 0.16 |
| San Isidro | 3.9 (0.6) | 257 (52.8) | 25.1 (3.2) | 4.7 (0.9) | 101.5 (41.8) |  |
| Señales | 4.0 (0.4) | 243 (37.8) | 25.0 (3.2) | 4.3 (0.7) | 69.3 (40.0) |  |
| Ponton | 3.6 (0.3) | 235 (50.2) | 25.0 (3.4) | 4.6 (0.9) | 90.6 (32.3) |  |
| San Glorio | 3.9 (0.4) | 223 (70.8) | 22.6 (3.2) | 4.5 (0.8) | 96.5 (38.1) | γ_ij_ = 1.47 |
| Total | 2.9 (0.9) | 247 (20.2) | 25.2 (3.3) | 5.5 (1.2) | 85.3 (31.1) |  |
