# Supplementary material for: Selective Pressures Explain Differences in Flower Color among Gentiana lutea Populations
Source: PLoS One. 2015 Jul 14;10(7):e0132522. doi: 10.1371/journal.pone.0132522 (PMC4501686; doi:10.1371/journal.pone.0132522)
Supplement: S1 Text — (DOC) [file pone.0132522.s005.doc]

**S1 Text. UV light reflectance**

Pollinators may detect UV light. If *Gentiana lutea* flowers emit in this range of the spectra, we may describe color considering the visible and UV light, unless there are no differences among populations or plants. If there are no differences among populations in the UV reflectance, we presume that UV light does not drive local adaptation; if yellow and orange flowering plants do not differ in the UV reflectance, we consider that selection driving yellow-orange color variation is not related to the UV light. In such cases a description of flower color variation from the human perspective fulfills our purpose: describing selection on the flower color variation seen by humans. To test for differences among populations and yellow-orange colors, we analyzed whether orange and yellow flowered-plants and populations differed in the UV reflectance.

We randomly selected 100 plants belonging to 9 populations. We measured the spectra with the same procedure as the one described in the methods section. To describe each plant UV reflectance, we considered the range from 300 to 400 nm, at 10 nm intervals (thus, each plant had its UV reflectance defined by eleven variates). By means of PCA, we reduced the number of variates. The first factor explained 75% of total variance, thus we used it in the following analysis. We run an analysis of variance with the first PCA component as the dependent variable, and qualitative color (orange or yellow) and population nested in color (since for the purpose of this analysis color was a qualitative trait), as independent factors. None of those factors were statistically significant (F_7,91_ = 1.77, *p* = 0.10; F _1,91_ = 1.54, *p* = 0.22; respectively for population and color), which indicates on the one hand that populations are similar in the UV reflectance, and that orange and yellow-flowered plants do not differ in the UV light.
